# Refractory Thrombectomy: Incidence and Related Factors in a Third-Level Stroke Treatment Center

**DOI:** 10.3390/jcm14238514

**Published:** 2025-11-30

**Authors:** Laura Restrepo-Carvajal, Francisco Hernández-Fernández, Angela Fernández-López, Laura Rojas-Bartolomé, Miguel de la Fuente, Cristian Alcahut, Blanca Serrano-Serrano, María Payá, Juan David Molina-Nuevo, Jorge García-García, Oscar Ayo-Martín, Rosa Angélica Barbella-Aponte, Gemma Serrano-Heras, Tomás Segura

**Affiliations:** 1 Neurology Department, General University Hospital of Albacete, 02006 Albacete, Spain; 2 Radiology Department, General University Hospital of Albacete, 02006 Albacete, Spain; 3 Radiology Department, General University Hospital of Cuenca, 16002 Cuenca, Spain; 4 Pathology Department, General University Hospital of Albacete, 02006 Albacete, Spain; 5 Research Unit, General University Hospital of Albacete, 02006 Albacete, Spain; 6 Cerebrovascular, Neurodegenerative, and Neuro-Oncological Diseases Group, Castilla-La Mancha Health Research Institute (IDISCAM), 13071 Albacete, Castilla-La Mancha, Spain

**Keywords:** ischemic stroke, mechanical thrombectomy, refractory thrombectomy, hyperdense artery sign

## Abstract

**Background/Objectives:** The efficacy of mechanical thrombectomy in large vessel occlusion has been established in multiple clinical trials as the standard of care for acute ischemic stroke. However, the incidence of refractory thrombectomy (RT), as well as related factors, remains unclear. **Methods**: We designed a retrospective study based on our prospective database from December 2014 to December 2023, collecting together the data on patients with large/medium vessel occlusion and reperfusion technique failure. We analyzed clinical, laboratory, histopathological, and radiological markers, as well as predictive variables of RT, mRS at three months, and symptomatic intracranial hemorrhage (sICH). **Results:** Among 733 patients, the incidence of refractory thrombectomy was 7.4% (n = 54). The most relevant causes were technical difficulties in passing through the occlusion (33.3%), clot resistance to extraction despite correct device placement (31.5%), and underlying intracranial stenosis (13%). A total of 20 recalcitrant thrombi were analyzed. Both atherotrombotic cause (OR 4.22, CI 95% 1.1–16.25; *p* = 0.04) and the absence of hyperdense artery sign (HAS) on CT (OR 0.26, 95% CI 0.09–0.79; *p* = 0.02) were independent predictors of RT outcome. For sICH, independent predictors were NIHSS at admission (OR 1.1; 95% CI 1.02–1.18; *p* = 0.008), protective effect in M1 occlusions (OR 0.28; 95% CI 0.09–0.88; *p* = 0.03), and prior IV thrombolysis (OR 0.38; 95% CI 0.16–0.93; *p* = 0.03). Finally, for favorable clinical outcome (mRS 0-2), a correlation was found with the NIHSS score at admission (OR 1.11; 95% CI 1.08–1.14; *p* = 0.0001), a history of diabetes (OR 0.53, 95% CI 1.02–1.18; *p*= 0.004), and RT status (OR 0.06, 95% CI 0.01–0.31; *p* = 0.001). No direct relationship was found between the clot composition and prognostic variables or first-line recanalization strategy. **Conclusions:** In our cohort, the incidence of refractory thrombectomies was low and associated with a worse clinical outcome. Absence of the hyperdense artery sign and the atherothrombotic stroke subtype were found to be independent predictors of refractory recanalization.

## 1. Introduction

The efficacy of mechanical thrombectomy (MT) in large vessel occlusion (LVO) has been established in multiple clinical trials as the standard of care in acute ischemic stroke (AIS). The absence of effective recanalization is a strong predictor of mortality, poor clinical prognosis, and symptomatic intracranial hemorrhage (sICH) [1]. However, there is a proportion of patients where the procedure is not successful (refractory thrombectomy, RT). According to different series, the incidence of RT varies from 8% to 30% [1,2,3]. However, its associated factors are not well stablished.

Regarding the definition of RT, in more recent studies [1,2,3], successful reperfusion efficacy is defined as modified ≥ TICI 2B. Although there is no clear consensus in the literature, the definition of RT can be considered as follows [2]: a presence of residual clots or an underlying pathology with rapid reocclusions, and the reocclusions occur within the first 72 h after a satisfactory MT.

The main causes of RT include technical difficulties in reaching the point of occlusion (vascular tortuosity, difficulty in arterial access, etc.) [4]; underlying pathologies (stenosis or intracranial dissections) [2,5,6]; and thrombi with high-fibrin composition or unusual compositions, such as calcium and septic thrombi [7,8].

There is a growing interest in studying the predictors of RT, with the aim of improving recanalization rates and clinical prognosis [9,10,11,12,13]. The main objective of our study was to analyze the incidence of RT in our series and to identify the main related clinical, laboratory, pathological, and radiological factors.

## 2. Materials and Methods

### 2.1. Design of the Study and Patient Selection

We conducted a retrospective, observational, and single-center study using a prospective database of patients with acute ischemic stroke treated with mechanical thrombectomy (MT) between December 2014 and December 2023. Those who met the criteria of RT were included in the study. RT was defined as follows: TICI < 2B after three passes, a presence of residual clots, or an unstable, underlying pathology with rapid reocclusions (or reocclusions within the first 72 h). We also included patients with TICI 2A (less than fifty per cent of the expected vascular territory) within the definition.

In our center, patients are selected for MT if they met the following criteria: (1) significant neurological deficit (generally NIHSS ≥ 6) and AIS with confirmed LVO or medium vessel occlusion in cerebral angiography; (2) the time from symptom onset to groin puncture is <24 h, including wake-up strokes and those of uncertain onset; (3) no intracranial hemorrhage on a non-contrast CT (NCCT); and (4) the presence of a ≥30% target mismatch profile on CT perfusion for patients with a time from symptom onset to EVT of >6 h. If no contraindication existed, prior treatment with intravenous recombinant tissue-type plasminogen activator (IVT) was administered prior to MT. The inclusion criteria for EVT did not change significantly during the study period (2014–2023).

### 2.2. Exclusion Criteria

1. Intracranial hemorrhage on non-contrast CT (NCCT).
2. Inaccessible distal arterial occlusions.
3. Effective recanalization after IVT.
4. Inability to revise the NCCT.
5. Absence of intracranial occlusion.

### 2.3. Clinical Data and Collected Variables

Demographic variables (age and sex) and clinical variables related to comorbidities and cardiovascular risk factors—including hypertension (HTN), diabetes mellitus (DM), hypercholesterolemia, coronary artery disease, peripheral artery disease, atrial fibrillation (AF), and previous anticoagulant treatment—were collected. Stroke etiology was assessed using the classic TOAST classification (Trial of ORG 10,172 in Acute Stroke Treatment Subtype Classification) [14]. The clinical evaluation was performed with the NIHSS scale on admission and the modified Rankin (mRS) scale at 3 months. A score of 0–2 was considered a favorable mRS. The following diagnostic–radiological variables were recorded: the Alberta Stroke Programme Early CT Score (ASPECTS) score on NCCT, the ratio of radiological mismatch, the hyperdense artery sign (HAS), and the arterial occlusion point. The laboratory markers analyzed were blood leukocytes, hematocrit, glycemia, and fibrinogen. The first-line recanalization strategy (stent retriever [SR], contact aspiration [CA], and a combined technique) was defined and presented in a dichotomized form. Additionally, cases requiring intracranial PTA/stenting as a rescue procedure were recorded. The type of clot extracted was classified according to our protocol described below. The response times were recorded and quantified in minutes. The influence of IVT and uncertain time evolution on different subgroups were analyzed. Procedural variables and anesthesia modality (local or general) were also recorded.

The causes of technique failure were classified into 6 groups. The interventionalist responsible for each case assigned the cause:

1. Technical difficulties (this category includes 3 subgroups):
  - Difficulties in reaching the point of occlusion (vascular tortuosity and difficulty in arterial access).
  - Target occlusion was reached but the operator was unable to pass the thrombus with the microwire/microcatheter. In these cases, no SR was deployed.
  - Target occlusion was reached and the SR was deployed but no reperfusion occurred after multiple retrievals (no clot retrieval or dislocation).
2. Underlying intracranial stenosis. A severe (70–99%) residual stenosis and/or in situ thrombosis with reocclusion on repeat angiographies.
3. Underlying intracranial dissection. The presence of a “double lumen” image; irregular segmental stenosis or “string sign”; fusiform or saccular dilatation (pseudoaneurysm); or a visible intimal flap (associated with compatible clinical signs) in the absence of other structural causes.
4. Clot hardness/burden. A resistance to extraction despite correct device placement and the concomitant existence of a predominant fibrinoplatelet or calcium clot.
5. Suboptimal devices. A poor adaptability to the consistency, size, or location of the thrombus. Poor apposition to the clot in stent retriever (SR), or an ineffective CA with suction catheters.
6. Severe intraprocedural complications. This category includes hemodynamic complications, early hemorrhagic transformation, vessel perforation, distal embolization, and iatrogenic dissection.

## 3. Neuroimaging Protocol

A complete multimodal CT was performed before MT. This included acquisition of NCCT, arterial occlusion assessment on an angio-CT scan, and a mismatch calculation by CT perfusion. All CT imaging was performed using a Philips Brilliance CT, 64-slice (Koninklijke Philips Electronics N.V., Amsterdam, The Netherlands). Signs of early ischemia were assessed on NCCT by the ASPECTS scale. A favorable penumbra profile was assessed when there was a mismatch of ≥30% between the mean transit time (MTT) and the cerebral blood volume (CBV) maps.

Moreover, 24 h after the procedure, or earlier in the case of unexpected neurological deterioration, a control NCCT was performed to assess the presence of established infarctions or sICH (defined as any intraparenchymal/subarachnoid hemorrhage associated with an increase in the NIHSS scale score at ≥4 points or death). All cases were initially assessed by one neuroradiologist and then reviewed by a second, blind independent neuroradiologist (reference value).

**Figure 1:**
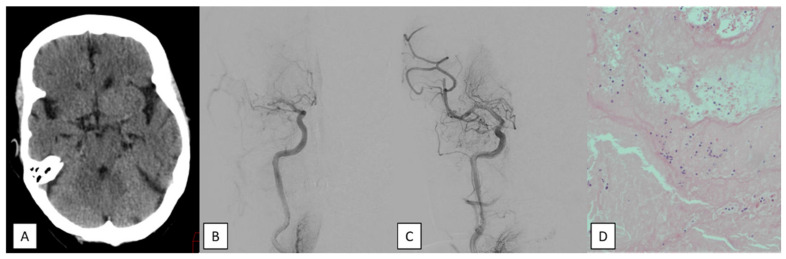
An 84-year-old female that was admitted with acute stroke with a NIHSS score of 19 points due to right M1 occlusion. (**A**) A non-contrast head CT that does not show a hyperdense vessel sign in the occluded right middle cerebral artery. (**B**) Digital subtraction angiography confirmed the right M1 occlusion. (**C**) Digital subtraction angiography after the forth pass with a stent retriever and stent–aspiration combination showing unsuccessful recanalization of the middle cerebral artery with clot migration into distal branches (TICI 2a). (**D**) HE20X predominance of platelets and fibrin, as well as scarce inflammatory cells and erithrocytes.

**Figure 2:**
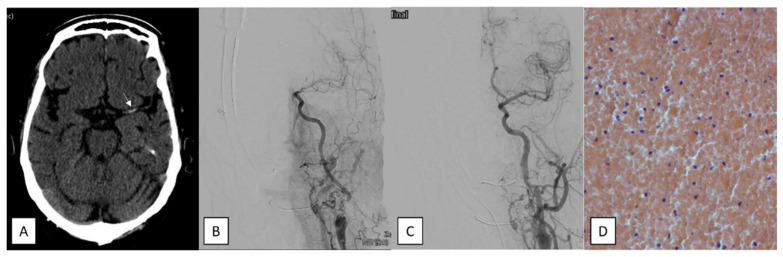
A 75-year-old female that was admitted with acute stroke with a NIHSS score of 11 points due to tandem occlusion (left proximal internal carotid artery stenosis and M1 occlusion). (**A**) A non-contrast head CT showing the left-sided hyperdense vessel sign in the middle cerebral artery (arrow). (**B**) Digital subtraction angiography confirmed the tandem occlusion. (**C**) Control angiogram showed complete recanalization of the middle cerebral artery with a TICI score of 3 after the first pass with stent retriever, as well as by showing concominant extracranial angioplasty and stenting of the proximal internal carotid artery lesion. (**D**) An obvious HE20x accumulation of erythrocytes with little inflammatory components and no fibrin and platelets.

The absence (Figure 1) or presence (Figure 2) of the HAS was classified based on visual inspection and according to the following criteria: (1) spontaneous visibility of the hyperdense occluded artery, (2) attenuation values of the occluded artery being greater than the ones of the surrounding parenchyma, (3) clot disappearance in the bone window, (4) unilaterality of this finding, and (5) an absence of subarachnoid hemorrhage.

## 4. Endovascular Procedure

Endovascular procedures were performed using a monoplane angiograph (Innova GE model General Electric Company, Boston, MA, USA) or a biplane angiograph (Azurion 7 B20/15 Koninklijke Philips Electronics N.V., Amsterdam, Netherlands). General anesthesia was systematically performed.

EVT were performed under the following general methodology:

Vascular access by puncture of the right femoral artery using the Seldinger technique. Placement of an 8 French femoral sheath.

Selective catheterization using hydrophilic 0.035″ guidewire and an 8F balloon-guided catheter (CGB) with placement in the cervical segment of the ICA in anterior circulation stroke.

Super selective catheterization of the occluded vessel and crossing of the occlusion with a 0.014″ microguidewire and a 0.021″ or 0.017″ microcatheter. Use of SR-assisted manual aspiration from the CGB with a 60 cc syringe; aspiration catheters connected to an automatic pump (CA) or combination of both techniques. CA was used systematically in occlusions of posterior circulation or as a second option in the anterior circulation. The combined technique (SR plus CA) was mainly used as a rescue treatment. After each pass, the device (intermediate catheter if withdrawn) and aspiration syringe were inspected for the presence of thrombus fragments. The device was gently washed with heparinized saline to remove thrombus fragments. Aspirated material was gently flushed with saline to identify any smaller fragments.

Angiographic control series in anteroposterior and lateral projections, as well as the determination of the TICI (thrombolysis in cerebral infarction) scale, were conducted.

## 5. Timeline of Technical and Technological Evolution

This study covered a long period of nine years, so various materials and techniques were used. Throughout the period, SR with CGB was mainly used as the technique of choice, exclusively employing 8F Flowgate CGB (Stryker Corporation, Kalamazoo, MI, USA) without exchange access. The microcatheters used did not vary substantially either, with Trevo Pro 0.021″ (Stryker Corporation, Kalamazoo, MI, USA) and Headway 0.017″ (MicroVention Inc., Tustin, CA, USA) being predominantly used. Likewise, the intracranial access microguide was exclusively a 0.014″ Traxcess microwire (MicroVention Inc., Tustin, CA, USA) throughout the study period.

The most used SRs as recanalization devices were Trevo XP 4 and 6 mm (Stryker Corporation, Kalamazoo, MI, USA); Solitaire FR 4 and 6 mm (Medtronic, Dublin, Ireland); Embotrap 5 mm (Johnson & Johnson, New Brunswick, NJ, USA); and Catch Mini 3 mm (Balt, Montmorency, France) for distal circulation. SRs that were later discontinued, such as Tigertriever 6 mm (Rapid Medical, Yokneam, Israel) and Revive SR 4 mm (Johnson & Johnson, New Brunswick, NJ, USA), were used occasionally. In 2020, Embotrap II was replaced by the new generation Embotrap III, and in 2022, Nimbus SR 4.5 began to be used for difficult thrombi.

When CA was used as a first-line strategy, 0.088″ Neuron MAX (Penumbra Inc., Alameda, CA, USA) and the Penumbra aspiration catheters (Penumbra Inc., Alameda, CA, USA) were used. Penumbra 3 MAX and 5 MAX were used until 2018, which is when they were then replaced by the Penumbra ACE line (0.060–0.068). Penumbra JET 7 (0.070″) was used from 2019 onwards.

Regarding intracranial PTA/stenting techniques, PTA was initially performed with 1.5-to-3 mm Gateway catheters (Stryker Corporation, Kalamazoo, MI, USA), and this was followed by the placement of 4-to-6 mm Solitaire AB stents. This strategy, starting from 2022, was replaced by NeuroSpeed double-lumen balloons (1.5–3.5 mm) with Credo stent (Acandis, Pforzheim, Germany) placement.

The cervical carotid recanalization techniques were sequential and systematically antegrade, including PTA and a placement of 7–9–40 mm X-act stents (Abbott laboratories, Chicago, IL, USA), throughout the study period.

Patients with wake-up and posterior circulation stroke were included from the onset of the period. In cases of wake-up stroke or an uncertain time of evolution, the patient was treated when they presented a favorable penumbra profile, as specified in the Neuroimaging protocol. Starting in 2020, the monoplane angiograph was replaced by the biplane angiograph. There has been no significant change in the anesthetic management strategy, with general anesthesia being used preferentially, except during the COVID-19 pandemic period (March–May 2020), which is when protocols had to be adapted and EVTs performed under local anesthesia.

Since 2021, in cases referred from our secondary centers where perfusion CT is not available, we abbreviated the protocol by performing only NCCT and angio-CT. Regarding the extent of the infarction, those with an ASPECTS score of <6 were excluded, in accordance with the clinical guidelines patent during the study period. Since 2021, systematic administration of IV tirofiban has been included when stent implantation was required.

The other general criteria for indication have not changed substantially at our center, as well as the exclusive administration of 0.9 mg/kg rt-PA as IVT.

## 6. Histopathological and Bacteriological Analysis

The thrombi were preserved in 4% formaldehyde and sent to the pathology department, which is where they were embedded in paraffin, sectioned at 4 μm, and then sectioned into 4 μm slices using a microtome. Consecutive sections were processed according to established histological and histochemical protocols, which included hematoxylin–eosin, periodic acid–Schiff (PAS), Gram, and Gomori’s Trichrome stains.

An expert neuropathologist conducted this study by analyzing the macroscopic and microscopic characteristics of the clots at 2× (scale bar 500 μm); 4× (scale bar 250 μm); 10× (scale bar 100 μm); and 20× (scale bar 50 μm) magnification. The pathologist then examined the thrombus sections stained with hematoxylin–eosin (H&E) under a light microscope to evaluate their composition, focusing on the following: red blood cells; white blood cells), including the number of polymorphonuclear cells and their status; the fibrin/platelet distribution; the degree of thrombus organization; and other components such as calcium, cholesterol crystals, fat, and the presence of bacteria. A semi-quantitative assessment was performed, as previously described by our group [15], through visual analysis using a method where an imaginary grid was overlaid on the slides to systematically analyze different regions of the thrombus. The clots were categorized into six types based on the following criteria.

- Red, fibrine, and mixed clots: A red clot occurs when the percentage of red blood cells is ≥60%. A fibrin-predominant clot is identified when the ratio of fibrin to platelet is ≥60%. A mixed clot is present if there is no clear predominance of these components.
- Septic clots: These occur when there is an increased number of white blood cells exhibiting morphological alterations. To detect bacteria, the slide was examined at 100× magnification (scale bar, 10 μm) using immersion oil.
- Calcium clots: a major component of calcified tissue.
- Fatty clots: an accumulation of adipose tissue as the major component.

## 7. Statistical Analysis

A descriptive study of the variables was performed using central and scattering tendency measures for quantitative variables and the exact calculation and percentage for qualitative variables. Normality of the sample was established using the Kolmogorov–Smirnov test. A comparison of the categorical variables between two or more subgroups was performed using the Pearson’s chi-squared test or, as applicable, the Fisher exact test. In addition to the RT status, we considered mortality, mRS at 3 months, and sICH as outcome variables. Mortality was defined as mRS = 6. HAS was determined in binary form (0 Absence HAS–1 Positive HAS), and the degree of interobserver agreement was calculated using the kappa index.

The comparisons between the quantitative variables were performed with the Student’s *t*-test. The comparisons between the medians was performed using the Wilcoxon signed-rank test, with the interquartile range (IQR) as a dispersion measure. A bivariate study was designed to examine the relationship between the main predictive variables and RT status. The independent effect of these variables was calculated, considering the presence of RT or sICH (bad outcome) as dependent variables, with multivariate binary logistic regression models. The odds ratio (OR) was calculated for each variable. The Hosmer–Lemeshow goodness-of-fit and sensibility tests were used to evaluate the global model fit. For favorable mRS (0–2), a proportional odds assumption was estimated using ordinal regression (parallel lines test). A significant result (*p* < 0.05) indicated that the assumption of parallel lines had been violated, suggesting that the effect of predictors had not been consistent across the ordinal outcome categories and that binary logistic regression had been carried out. The variables included in the multivariate analysis, in addition to the age and NIHSS at admission, were those with statistical significance *p* < 0.1 in the bivariate models. A *p* < 0.05 was considered significant for all analyses. All of the results were analyzed with the statistical software IBM SPSS Statistics Version 26 (SPSS, Chicago, IL, USA).

## 8. Ethical Approval

The studies involving humans received approval from the Albacete University Hospital Ethics Committee (date and reference number (2019/03/031)). Good clinical practice guidelines and the guidelines detailed in the declaration of Helsinki were followed. Confidentiality of the data obtained was kept and they were only used for study purposes. The studies were conducted in accordance with the local legislation and institutional requirements. The participants provided their written informed consent to participate in this study. Written informed consent was obtained from the individual(s) consent form for participation, which was distributed to all participants and signed for the publication (indicating their agreement to take part in the experiment and any potentially identifiable images or data included in this article).

## 9. Results

A total of 733 MTs were performed in the period described. Of those, 679 were effective (ET, 92.6%) and 54 were RT (7.4%), given by the TICI outcomes of 0–2 A. Among all of the 733 patients who underwent MT, 407 (56%) had a favorable mRS score 3 months after the procedure.

The mean values of the main clinical, laboratory, histopathological, and radiological variables analyzed are described in Table 1.

There were no significant differences between age/sex and the presence of RT or ET. A higher prevalence of DM was observed in the RT group with respect to ET (40.7% vs. 27.2%, *p* = 0.04). The atherothrombotic cause was also more prevalent in the RT group (38.9% vs. 19.7%, *p* = 0.003). As long as M1 occlusions had a greater impact on the group of ET (37.1% vs. 25.9%, *p* = 0.026), PCA and ACA occlusions were more frequent in the RT group (Table 1).

The radiological findings of HAS on NCCT was more frequent in the ET patient group (59.5% vs. 31.5%, *p* < 0.0001). The interrater agreements were excellent for HAS detection (*p* < 0.0001, weighted Cohen’s kappa, 0.81). No relationship was found between the hematocrit data of the emergency samples and the presence of SAH (*p* = 0.71) or RT (*p* = 0.11).

Regarding the first-line recanalization strategy, patients treated with SR were more likely to achieve ET (80.8% vs. 65%, *p* = 0.02), while patients had a higher incidence of RT in whom CA was selected as the first-line technique (35% vs. 15.8%, *p* = 0.004). A total of 34 patients (4.6% of the total) required intracranial PTA/stenting as a rescue procedure, which was more frequent in the RT group (14.8% vs. 3.8%, *p* = 0.002).

With respect to the histopathological analyses of clots, it should be noted that patients with RT results had a smaller number of analyzable samples. Suitable thrombotic material could be extracted and analyzed in more than one-third of refractory patients (37.7%, 20 cases). Specifically, 1 red clot, 9 fibrin-rich clots, and 7 mixed clots were extracted. The rest were a miscellany of atypical clots: 1 septic, 1 fatty, and 2 calcium. In this subgroup of patients, calcium clots had a higher incidence (10% vs. 1.1%, *p* = 0.03). On the other hand, within the ET group, there was only 14.3% of cases (i.e., 97) without an evaluable sample (*p* = 0.0001). The histopathological finding of red clots was more frequent in the ET group (21.8% vs. 1.9%, *p* < 0.0001).

Among the main causes of RT were technical difficulties in crossing the occlusion (33.3%), recalcitrant clots (31.5%), and underlying intracranial stenosis (13%) (Table 2).

Subsequently, multivariate analyses were performed (Table 3).

The multivariate analysis of RT status showed the following independent factors: atherotrombotic origin (OR 4.22, 95% CI 1.1–16.2; *p* = 0.04) and absence of HAS (OR 0.26, 95% CI 0.09–0.79; *p* = 0.02). The Hosmer–Lemeshow test resulted in an Χ^2^ value = 4.35 (df = 8, *p* = 0.82). The model correctly classified 96.5% of patients with RT. The significance in the omnibus test in the model was *p* = 0.001. The R-square values of Cox and Snell—and the R square of Nagelkerke—were 0.07 and 0.26, respectively.

For sICH, logistic regression showed an independent effect with NIHSS at admission (OR 1.1; 95% CI 1.02–1.18; *p* = 0.008). A protective effect was found when arterial occlusion was in M1 (OR 0.28; 95% CI 0.09–0.88; *p* = 0.03) and when prior IV thrombolysis was administrated (OR 0.38; 95% CI 0.16–0.93; *p* = 0.03). The Hosmer–Lemeshow test resulted in an Χ^2^ value = 11.5 (df = 8, *p* = 0.17). The model correctly classified 96% of patients with sICH. The significance in the omnibus test in the model was *p* = 0.041. The R-square values of Cox and Snell—and the R square of Nagelkerke—were 0.04 and 0.15, respectively.

Finally, to estimate the relationship between these variables and favorable clinical prognosis (mRS 0-2), ordinal regression (parallel lines test) showed a *p* = 0.0001, so the proportional assumption was considered violated. Logistic regression showed a correlation with the NIHSS score at admission (OR 1.11; 95% CI 1.08–1.14; *p* = 0.0001), a history of DM (OR 0.53, 95% CI 1.02–1.18; *p* = 0.004), and RT status (OR 0.06, 95% CI 0.01–0.31; *p* = 0.001). The Hosmer–Lemeshow test resulted in an Χ^2^ value = 8.9 (df = 8, *p* = 0.34). The model correctly classified 60% of patients with mRS 0-2. The significance in the omnibus test in the model was *p* = 0.0001. The R-square values of Cox and Snell—and the R square of Nagelkerke were 0.21 and 0.16—respectively.

The remaining variables showed no significant relationship with the prognostic outcome variables (Table 3).

A subanalysis of the relationship between the clot composition and the first-line strategy for revascularization showed no statistical differences (Table 4).

## 10. Discussion

The term refractory thrombectomy (RT) is an increasingly used definition in neurovascular groups since it is associated with high rates of complications and poor clinical prognosis [1,2,3,4,5]. Refractoriness incidence in our series was 7.4%, which is similar to other series published [2] but less than the first positive MT randomized trials [16]. This could be due to an improvement in AIS care times, improved protocols in MT, technological advances, and new devices.

In our study, the RT group was associated with higher mRS at 3 months, higher mortality, and higher incidence of sICH. Lower NIHSS scores, consistent with previous work [11], have been associated with a better clinical outcome. Our first refractoriness cause valued by neurointerventionists was technical difficulty in crossing the occlusion (33%), which is consistent with other similar studies and is widely recognized as the main problem in these procedures [7].

The second most frequent cause was clot hardness/burden (31%). Numerous studies correlate clot hardness and predominant fibrine composition with worse angiographic outcome [17,18,19]. On the contrary, red clots (formed mainly by red blood cells), have been related to better angiographic results, probably due to their lower coefficient of friction [20,21,22,23,24]. Our study shows that red clots were more common in the ET group and calcium clots were more prevalent in the RT group. Moreover, cases that were treated with SR as the first-line strategy were more prone to achieve ET (80.8% vs. 65%, *p* = 0.02), and the opposite was true for CA—which had higher RT rates (35% vs. 15.8%, *p* = 0.004). As a matter of course, rescue PTA/stenting was more prevalent in the RT group (14.8% vs. 3.8%, *p* = 0.002). However, no correlation was observed between the clot composition and prognostic variables or first-line recanalization strategy.

Interestingly, some studies have identified HAS as a radiological marker of red thrombi in NCCT prior to MT, resulting in better functional outcomes at 3 months [25]. Others, however, have found no association of this radiologic sign with functional outcome [26,27,28].

In this series, HAS on NCCT was more frequent in the ET group (59.5% vs. 31.5%, *p* = 0.0001), and its absence was an independent indicator of RT (OR 0.26, 95% CI 0.09–0.79; *p* = 0.02). In addition, the interrater agreements were excellent for HAS detection (*p* < 0.0001, weighted Cohen’s kappa, 0.81). Despite the controversy surrounding the relationship between HAS and RT, our data are in line with recent studies that have presented results suggesting HAS as a potential pre-procedure indicator of challenging clots and worse clinical outcomes [29].

The other factor we have found to be associated with RT is the atherothrombotic origin of stroke (OR 4.22, CI 95% 1.1–16.25; *p* = 0.04). In fact, underlying intracranial stenosis was the main difficulty factor in 13% of our patients. This result can be explained by a greater association of intracranial atheromatous stenoses and tandem occlusions with reocclusions and RT [30,31]. More specifically, recurrent AIS or reocclusions after EVT for atherothrombotic stroke-related causes are especially higher in intracranial stenosis [32].

Additionally, the bivariate study found a significant relationship between RT and DM (*p* = 0.04). Although this was not confirmed in the multivariate study, it was demonstrated to be an independent factor for poor clinical prognosis (OR 0.53, 95% CI 1.02–1.18; *p* = 0.004). In addition to the well-known relationship between DM and extra and intracranial atheromatosis, this factor relates specifically to RT [2,31]. In diabetic patients, fibrosis and vascular remodeling produce higher intracranial plaque burden, there is a greater degree of stenosis, and there is also a greater inflammatory enhancement in specific segments of intracranial arteries compared to non-diabetic patients [33,34].

In our study, IVT was significantly associated with a lower risk of sICH (OR 0.38; 95% CI 0.16–0.93; *p* = 0.03). Caution should be exercised when interpreting this result since the treatment protocol during the period study (2014–2023) excluded patients with low ASPECTS scores. Therefore, the subjects showed relatively small core infarct of the patients at the time of admission (mean ASPECTS = 9, mismatch average ratio >80%). Although there may be safety concerns in the subgroup of patients with large infarcts [35], systematic studies have associated IVT with better recanalization rates without an increase in sICH rates after MT [36]. Another factor associated with lower rates of sICH in our study were M1 occlusions (OR 0.28; 95% CI 0.09–0.88; *p* = 0.03). Despite some reports suggesting that M1 occlusions have a significant risk of sICH [37], the use of second- and third-generation devices together with the high ET rates in this segment in our series (91.1%) may explain the results.

This study has some limitations, especially since it was carried out in a single center and due to its retrospective nature. The long study period and the ongoing evolution of treatments and devices may have affected the internal consistency of the work. Our database is not designed to distinguish the dominance of M2 occlusions; as such, to a large extent, the analysis of LVO and medium vessel occlusions was performed in aggregate. Endovascular techniques and devices were heterogeneous according to operator discretion. The accuracy of HAS assessment could have been improved by systematically measuring Hounsfield Units. However, we collected this data systematically until 2021 [15], and the lack of correlation with clot composition led us to abandon it as a marker of refractoriness. Other published studies suggest the same conclusion [25]. Although patients with RT have lower histopathological study rates, we have been able to analyze a substantial number of cases (n = 20). Despite these limitations, our study had a large cohort from the real world at his disposal (n = 733), a comprehensive prospective database, and broad anatomopathological studies, which enabled associative studies of the variables to be carried out.

In conclusion, we confirmed atherothrombotic stroke and the absence of SAH as predictive factors for RT. Further studies are needed on the histological characteristics of the thrombus and its clinical and radiological parameters before treatment, which would allow us to guide the reperfusion strategy in patients at high risk of RT.

## Figures and Tables

**Table 1:** The baseline characteristics of the sample. Comparative bivariate analysis of the effective thrombectomy (ET) and refractory thrombectomy (RT) groups. ACA: anterior cerebral artery; AF: atrial fibrillation; CA: contact aspiration; DM: diabetes mellitus; HAS: hyperdense artery sign; HBP: high blood pressure; IQR: interquartile range; N: absolute frequency; NIHSS: National Institutes of Health Stroke Scale; mRS: modified Rankin scale; PCA: posterior carotid artery; PTA: percutaneous transluminal angioplasty; sICH: symptomatic intracranial hemorrhage; SR: stent retriever; SD: standard deviation; and TICA: terminal carotid artery.

	ET N = 679 (92.6%)	RT N = 54 (7.4%)	*p* Value
**Demographics and Comorbidities**			
Age, Mean Years (±SD)	70.2 (12.7)	71.8 (11.2)	0.3
Sex Female, N (%)	295 (43.4)	22 (40.7)	0.77
HBP, N (%)	464 (68.3)	38 (70.4)	0.88
DM, N (%)	185 (27.2)	22 (40.7)	**0.04**
Hypercholesterolemia, N (%)	282 (41.5)	23 (42.6)	0.89
Ischemic Heart Disease, N (%)	77 (11.3)	8 (14.8)	0.5
Intermittent Claudication N (%)	22 (3.2)	2 (3.7)	0.91
AF, N (%)	273 (40.4)	19 (35.2)	0.47
Anticoagulation, N (%)	164 (24.2)	37 (68.5)	0.15
**Laboratory Findings**			
Glycemia, Mean gr/dL (±SD)	138.9 (53.2)	142.5 (52.2)	0.6
Leukocytes, Mean/μL (±SD)	9576 (3312.4)	10,946 (6637.9)	0.14
Fibrinogen, Mean mg/dL (±SD)	361.85 (86.3)	364.35 (85.2)	0.84
Hematocrit, Mean (%)	41.9	40.6	0.11
**Stroke Characteristics**			
Baseline NIHSS, Median (IQR)	17 (11–23)	16.5 (11–22)	0.85
Territory occluded, N (%)			
M1	272 (37.1)	14 (25.9)	**0.026**
M2	101 (14.9)	7 (13)	0.44
TICA	73 (10.8)	7 (13)	0.37
Carotid tandem	112 (16.5)	8 (14.8)	0.47
ACA	4 (0.6)	3 (5.6)	**0.011**
Basilar	46 (6.8)	5 (9.3)	0.32
PCA	18 (2.7)	6 (11.1)	**0.006**
HAS, N (%)	404 (59.5)	17 (31.5)	**0.0001**
ASPECTS score, Median (IQR)	9 (8–10)	9 (8–10)	0.33
Mismatch, Average Ratio (±DE)	82 (20.2)	85.3 (15.4)	0.23
IV Thrombolysis, N (%)	219 (32.3)	11 (20.4)	0.09
Uncertain time evolution	195 (28.7)	11 (20.4)	0.21
Median Time from Symptom Onset to Groin Puncture, Minutes (IQR)	295 (201–577)	283 (226.5–465)	0.99
Local anesthesia, N (%)	7 (13)	39 (5.7)	0.071
First-line strategy, N (%)			
SR	523 (80.8)	26 (65)	**0.02**
CA	102 (15.8)	14 (35)	**0.004**
Combined technique	22 (3.4)	0 (0)	0.63
Intracranial PTA/stenting rescue	26 (3.8)	8 (14.8)	**0.002**
Atherothrombotic Cause, N (%)	134 (19.7)	21 (38.9)	**0.003**
Cardioembolic Cause, N (%)	351 (51.7)	19 (35.2)	**0.02**
Intracranial Stenosis, N (%)	47 (6.9)	7 (13)	0.11
**Histopathological analysis**			
Red clot, N (%)	148 (26.5)	1 (5)	**0.0001**
Fibrin clot, N (%)	263 (47)	8 (40)	0.65
Mixed clot, N (%)	124 (22.2)	7 (35)	0.18
Septic clot, N (%)	16 (2.9)	1 (5)	0.45
Calcium clot, N (%)	6 (1.1)	2 (10)	**0.03**
Fatty clot, N (%)	2 (0.4)	1 (5)	0.1
**Outcome Variables**			
Favorable mRS at 3 Months, N (%)	407 (59.9)	5 (9.3)	**0.0001**
sICH, N (%)	24 (3.5)	7 (13)	**0.005**
Mortality, N (%)	91 (13.4)	22 (40.7)	**0.0001**

**Table 2:** Causes of refractory thrombectomy. N: absolute frequency; %: percentage.

	N	%
Technical Difficulties	18	33.3
Underlying Intracranial Stenosis	7	13
Thrombus Hardness/Burden (Recalcitrant Clot)	17	31.5
Suboptimal Devices	4	7.4
Severe Intraprocedural Complication	8	14.8
Intracranial Dissections	0	0
Total	54	100

**Table 3:** Multivariate analysis. Binary logistic regression for refractory thrombectomy (RT), symptomatic intracranial hemorrhage (sICH), and favorable clinical outcome (mRS 0-2). ACA: anterior cerebral artery; CA: contact aspiration; CI: confidence interval; DM: diabetes mellitus; HAS: hyperdense artery sign; IVT: intravenous thrombolysis; NIHSS: National health Institute Stroke Scale; N/A: not applicable and OR: odds ratio.

Variables	Outcomes	OR	95% CI for OR		*p* Value
			Inferior	Superior	
**Age**	RT	0.98	0.94	1.03	0.44
	sICH	1.02	0.98	1.06	0.31
	mRS 0-2	1.01	0.36	0.83	0.09
**M1 occlusion**	RT	0.47	0.15	1.5	0.20
	sICH	0.28	0.08	0.88	**0.03**
	mRS 0-2	0.83	0.55	1.24	035
**ACA occlusion**	RT	59,111	0	-	1
	sICH	0	0	0	0.99
	mRS 0-2	0	0	0	0.99
**ACP occlusion**	RT	0.11	0.006	1.4	0.09
	sICH	0	0	0	0.99
	mRS 0-2	0.28	0.74	11.01	0.13
**NIHSS**	RT	0.95	0.88	1.02	0.15
	sICH	1.1	1.02	1.18	**0.008**
	mRS 0-2	1.11	1.08	1.14	**0.0001**
**DM**	RT	1.11	0.39	3.2	0.84
	sICH	0.53	0.21	1.32	0.17
	mRS 0-2	0.55	0.36	0.83	**0.004**
**IVT**	RT	0.57	0.18	1.94	0.58
	sICH	0.38	0.16	0.93	**0.03**
	mRS 0-2	1.06	0.72	1.58	0.75
**Local anesthesia**	RT	0.24	0.05	1.11	0.07
	sICH	1.24	0.14	11.36	0.85
	mRS 0-2	1.48	0.6	3.66	0.4
**SR as first-line**	RT	15,328	0	-	1
	sICH	0.8	0.09	6.9	0.84
	mRS 0-2	1.05	0.4	2.78	0.92
**CA as first-line**	RT	1693	0	-	1
	sICH	2.53	0.2	32.63	0.48
	mRS 0-2	1.31	0.45	3.8	0.62
**Red clot**	RT	0.19	0.02	1.52	0.12
	sICH	1.41	0.48	4.11	0.53
	mRS 0-2	1.31	0.85	2.01	0.23
**Calcium clot**	RT	0.7	0.01	5.03	0.72
	sICH	2.2	0.19	25.17	0.52
	mRS 0-2	1.27	0.26	6.22	0.76
**Atherothrombotic Cause**	RT	4.22	1.10	16.25	**0.04**
	sICH	0.46	0.13	1.6	0.22
	mRS 0-2	0.84	0.47	1.51	0.56
**Cardioembolic Cause**	RT	0.43	0.11	1.64	0.22
	sICH	0.92	0.3	2.83	0.88
	mRS 0-2	1.07	0.68	1.67	0.77
**HAS**	RT	0.26	0.09	0.79	**0.02**
	sICH	1.55	0.21	1.32	0.36
	mRS 0-2	1.37	0.91	2.05	0.13
**RT**	RT	N/A	N/A	N/A	N/A
	sICH	0.44	0.08	2.44	0.35
	mRS 0-2	0.06	0.01	0.31	**0.001**

**Table 4:** Correlations between the first-line strategy recanalization and the anatomopathological types of clots. CA: contact aspiration; N: absolute frequency; and SR: stent retriever.

	Red		Fibrin		Mixed		Septic		Fatty		Calcium	
	N (%)	*p*	N (%)	*p*	N (%)	*p*	N (%)	*p*	N (%)	*p*	N (%)	*p*
**First-line strategy: SR**	118 (79.2)	0.39	222 (81.9)	0.91	112 (85.5)	0.25	12 (75)	0.51	1 (33.3)	0.09	7 (87.5)	1
**First-line strategy: CA**	24 (16.1)	0.69	42 (15.5)	0.72	14 (16.7)	0.16	3 (18.8)	0.71	2 (66.7)	0.059	1 (12.5)	1

## Data Availability

Data analysis was made by F.H.-F. and L.R.-C. The raw data supporting the conclusions of this article will be made available by the authors, without undue reservation.

## Abbreviations

AIS	Acute ischemic stroke
AF	Atrial fibrillation
ASPECTS	Alberta Stroke Program Early Computed Tomography Score
CGB	Balloon-guided catheter
CBV	Cerebral blood volume
CA	Contact aspiration
DM	Diabetes mellitus
ET	Effective thrombectomy
EVT	Endovascular treatment
FPE	First pass effect
HBP	High blood pressure
HAS	Hyperdense artery sign
ICH	Intracranial hemorrhage
IVT	Intravenous thrombolysis
LVO	Large vessel occlusion
MT	Mechanical thrombectomy
MTT	Mean transit time
mRS	Modified Rankin scale
NCCT	Non-contrast computed tomography
PTA	Percutaneous transluminal angioplasty
RT	Refractory thrombectomy
SR	Stent retriever
TICA	Terminal carotid artery
TICI	Thrombolysis in cerebral infarction scale
